# Safety study of live, oral human rotavirus vaccine: A cohort study in United States health insurance plans

**DOI:** 10.1080/21645515.2018.1450123

**Published:** 2018-04-13

**Authors:** Veena Hoffman, Remon Abu-Elyazeed, Cheryl Enger, Daina B. Esposito, Michael C. Doherty, Scott C. Quinlan, Kathleen Skerry, Crystal N. Holick, Peter Basile, Leonard R. Friedland, Nicolas Praet, Stéphanie Wéry, Corinne Willame, David D. Dore, Dominique Rosillon

**Affiliations:** a Optum Epidemiology, Ann Arbor, MI, US; b GSK, Philadelphia, PA, US; c HealthCore Inc., Andover, MA, US; d Optum Epidemiology, Boston, MA, US; e HealthCore Inc., Alexandria, VA, US; f HealthCore Inc., Wilmington, DE, US; g GSK, Wavre, Belgium

**Keywords:** rotavirus vaccine, safety, health insurance claims data, intussusception, acute lower respiratory tract infection, Kawasaki disease, convulsion, mortality

## Abstract

As part of a regulatory commitment for post-licensure safety monitoring of live, oral human rotavirus vaccine (RV1), this study compared the incidence rates (IR) of intussusception, acute lower respiratory tract infection (LRTI) hospitalization, Kawasaki disease, convulsion, and mortality in RV1 recipients versus inactivated poliovirus vaccine (IPV) recipients in concurrent (cIPV) and recent historical (hIPV) comparison cohorts. Vaccine recipients were identified in 2 claims databases from August 2008 – June 2013 (RV1 and cIPV) and January 2004 – July 2008 (hIPV). Outcomes were identified in the 0–59 days following the first 2 vaccine doses. Intussusception, Kawasaki disease, and convulsion were confirmed via medical record review. Outcome IRs were estimated. Incidence rate ratios (IRRs) were obtained from Poisson regression models. A post-hoc self-controlled case series (SCCS) analysis compared convulsion IRs in a 0–7 day post-vaccination period to a 15–30 day post-vaccination period. We identified 57,931 RV1, 173,384 cIPV, and 159,344 hIPV recipients. No increased risks for intussusception, LRTI, Kawasaki disease, or mortality were observed. The convulsion IRRs were elevated following RV1 Dose 1 (cIPV: 2.07, 95% confidence interval [CI]: 1.27 – 3.38; hIPV: 2.05, 95% CI: 1.24 – 3.38), a finding which is inconclusive as it was observed in only one of the claims databases. The IRR following RV1 Dose 1 in the SCCS analysis lacked precision (2.40, 95% CI: 0.73 – 7.86). No increased convulsion risk was observed following RV1 Dose 2. Overall, this study supports the favorable safety profile of RV1. Continued monitoring for safety signals through routine surveillance is needed to ensure vaccine safety.

## Introduction

Rotavirus is the most common cause of acute or severe gastroenteritis in children younger than 5 years of age. In 2008, the virus was associated with an estimated 453,000 deaths worldwide each year.^1^ As of 2009, rotavirus caused an estimated 3 million cases of diarrhea among children in the United States (US) each year with medical attention sought for 500,000 children and resulting in 60,000–70,000 hospitalizations.^2^

Two rotavirus vaccines are currently licensed by the US Food and Drug Administration. Human-bovine reassortant rotavirus vaccine (RV5, RotaTeq, Merck and Co., Inc., USA), was licensed in the US in 2006 and is administered in 3 doses at 2, 4, and 6 months of age. Live, oral human rotavirus vaccine (RV1, Rotarix, GSK, Belgium) was licensed in the US in 2008 and is administered in 2 doses at 2 and 4 months of age. Pre-licensure clinical studies of both RV5 and RV1 demonstrated high efficacy and safety profiles.^3-5^ RV1 is licensed in over 130 countries worldwide including US, Canada, Mexico, Australia, and countries of the European Union. Since the implementation of the rotavirus vaccination program in the US, significant declines in rotavirus illness and associated emergency department visits and hospitalizations have occurred.^6-9^

The first oral rhesus-human reassortant rotavirus vaccine (RRV-TV, Rotashield, Wyeth Laboratories, USA) was licensed in the US in October 1998 and voluntarily withdrawn from the market 9 months later because of its association with the occurrence of intussusception.^10^^,^^11^ Intussusception is a rare form of intestinal obstruction in which a segment of the bowel prolapses into a more distal portion. Most cases occur in infants who are <12 months of age.^12-14^ The baseline rate of intussusception in the US population during the first year of life was estimated at 34 per 100,000 infant-years from 2002 – 2004.^15^ The underlying cause of intussusception is unknown, but it has been associated with several pathogens, including adenoviruses.^16^

RV1 was not associated with an increased risk of intussusception or other safety events in pre-licensure clinical trials.^4^^,^^5^ During the early post-approval period, studies based on spontaneous adverse event reporting and observational studies demonstrated a favorable safety profile for RV5.^17-20^ However, at the time these early post-approval studies of RV5 safety were conducted, a sufficient number of RV1 doses were not yet administered to assess the risk of intussusception and other safety events. This study was designed as part of a regulatory commitment for the post-licensure safety monitoring of RV1, with the objective of assessing the safety profile of RV1 starting immediately upon the 2008 launch of RV1 in the US in a population of infants with commercial health insurance who would not be represented in active safety surveillance systems such as the Vaccine Safety Datalink. Specifically, this study estimated the risk of several safety outcomes, including intussusception, hospitalization due to acute lower respiratory tract infection (LRTI), Kawasaki disease, convulsion, and all-cause mortality, among infants receiving RV1 under routine conditions compared to infants receiving inactivated poliovirus vaccine (IPV).

## Methods

### Data sources

This phase IV observational cohort study (NCT00875641) was conducted within the Optum Research Database (ORD) and the HealthCore Integrated Research Database (HIRD). These databases contain health insurance claims information from large, commercially insured populations in the US. For members with insurance coverage in the participating health plans, the databases include demographics and pharmacy, medical, and facility claims which are submitted by providers for reimbursement of healthcare services and provide dates of services, procedures, and their accompanying diagnoses dating back to 1993 (ORD) and 2004 (HIRD). In both databases, diagnoses are recorded using International Classification of Diseases, 9th Revision (ICD-9) codes and International Classification of Diseases, 10th revision (ICD-10) codes. Medical procedures, including vaccine administrations, are coded using ICD-9 procedure, ICD-10 procedure, Common Procedural Terminology (CPT), and Health Care Financing Agency Common Procedure Coding System codes. Medications are identified by National Drug Classification codes. ICD-9 diagnosis and procedure codes are recorded in both databases until the date of implementation of ICD-10 coding in the US (01 October 2015).

Mortality information was collected via external linkage to the National Death Index (NDI), in addition to the claims databases. The NDI database is a central computerized index of death record information comprised of data on file in the state vital statistics offices.^21^ The National Center for Health Statistics maintains the database which contains both date and cause of death for adults and children.

### Study population

Infants receiving at least one dose of RV1 as part of routine health care were identified within the ORD and HIRD prospectively from 01 August 2008 – 30 June 2013. Two comparison groups of infants receiving at least one dose of IPV were also identified: 1) IPV recipients identified prospectively during the same time frame as the RV1 recipients (concurrent IPV [cIPV] recipients), and 2) IPV recipients identified retrospectively from 01 January 2004 – 31 July 2008 (recent historical IPV [hIPV] recipients). IPV recipients were chosen for the comparison groups as IPV is part of the recommended immunization schedule for infants in the US and is administered on the same routine visit as RV1. As it was expected that most cIPV recipients would have received RV5 per routine vaccination recommendations, the group of hIPV recipients was formed in order to have a comparator group who did not receive any rotavirus vaccination. All infants were required to be enrolled in the participating health insurance plans within 30 days of birth, to remain continuously enrolled until the time of cohort entry, and to be younger than one year of age at the time of entry into the study population. RV1 recipients were required to have no previous dose of RV5 prior to or concurrent with the first RV1 dose. Recipients of cIPV were required to have no previous or concurrent dose of RV1. Recipients of hIPV were required to have no previous or concurrent dose of RV1 or RV5. RV1, RV5, and IPV administrations were identified on the basis of CPT codes recorded on the claims submitted by medical providers.

The IPV recipients were frequency-matched to the RV1 recipients in a 3:1 ratio by age (in months) at first vaccination, gender and calendar quarter of vaccination (within the same year for cIPV recipients). Based on the expected age distribution of intussusception cases in the first year of life,^15^ matching by age in months was expected to provide sufficient control for age effects.

### Identification of safety outcomes

Cases of intussusception, hospitalization due to acute LRTI, Kawasaki disease, convulsion, and all-cause mortality were identified from the health insurance claims using International Classification of Diseases, 9^th^ Revision (ICD-9) diagnosis codes associated with inpatient, outpatient, or emergency department (ED) encounters and/or current procedural terminology (CPT) or ICD-9 procedure codes (Table 1). For intussusception, acute LRTI, and convulsion, multiple events per patient were considered if the diagnosis and/or procedure codes were indicative of separate events, rather than part of the evaluation or follow-up for a single event. For Kawasaki disease, only the first event was included in the analysis. Claims-identified cases of intussusception, Kawasaki disease, and convulsion were confirmed through review of medical records corresponding to the relevant billed medical service. Medical record confirmation of intussusception, Kawasaki disease, and convulsion was conducted by independent adjudication panels. Each adjudication panel consisted of 3 clinicians who reviewed the abstracted medical records, which were blinded with respect to identifying information and vaccine exposure, to determine whether the case definition criteria for each study outcome were fulfilled. Case definition criteria were consistent with definitions from the Brighton Collaboration Working Group for intussusception and the Brighton Collaboration Seizure Working Group for convulsion (Table 1). Case definition criteria for Kawasaki disease were based on US Centers for Disease Control and Prevention surveillance definitions. For all medical record-reviewed outcomes, the date of case confirmation was the date of the diagnosis recorded in the medical records. To capture deaths which may not have a corresponding health insurance claim, we also identified deaths via linkage of the claims data to the NDI.

**Table 1.** Pre-specified study outcome definitions.

Outcome	Code(s)	Risk Period(s)	Medical Record Confirmation Criteria
Intussusception^*^	ICD-9 diagnosis code 543.9, 560.0 or CPT code 74283	0–59	Level 1 definite criteria per the Brighton Collaboration (radiographic or surgical confirmation, or evidence at autopsy)
		0–6	
		0–29	
Hospitalization due to acute LRTI^†^	ICD-9 diagnosis code 466, 480–486, 487.0, 490, 513.0;	0–59	N/A
	AND	0–6	
	At least one procedure code for radiologic examination of the chest within +/- 3 days of the diagnosis: CPT 71010, 71015, 71020 – 71023, 71030, 71034, 71035; ICD-9 procedure 87.44, 87.49	0–29	
Kawasaki Disease^*^	ICD-9 diagnosis code 446.1	0–59	US CDC surveillance case definition: Fever ≥5 days duration (or until administration of IV immunoglobulin), AND presence of at least 4 of the following clinical signs: rash, cervical lymphadenopathy(at least 1.5 cm in diameter), bilateral conjunctival injection, oral mucosal changes, peripheral extremity changes
Convulsion^*^	ICD-9 diagnosis code 333.2, 345, 779.0, 780.3	0–59	Brighton Collaboration Seizure Working Group definition: Generalized or focal tonic, clonic, tonic-clonic, or atonic motor manifestations, AND witnessed sudden alteration of consciousness (Level 1 of diagnostic certainty) OR history of altered consciousness (Level 2 of diagnostic certainty)
All-Cause Mortality^‡^	ICD-9 diagnosis code 798, 798.0, 798.1, 798.2, 798.9; UB-92 form patient discharge status code 20, 40, 41, 42	0–59	N/A
	Additional deaths identified through the NDI data		

Abbreviations: CDC = Centers for Disease Control and Prevention; CPT = current procedural terminology code; ED = emergency department; ICD-9 = International Classification of Diseases, 9th Revision; LRTI = lower respiratory tract infection; N/A = not applicable; NDI = National Death Index; UB = Uniform/Universal billing; US = United States.

* Codes were associated with a hospitalization or ED encounter.

† Codes were associated with a hospitalization.

‡ Codes were associated with a hospitalization, ED, or outpatient encounter.

### Follow-up period

All outcomes were assessed in a 0–59 day risk period following each of the first 2 doses of RV1 or IPV administration (up to a total of 120 days of follow-up). This post-vaccination risk period, which is the expected interval between the first and second RV1 doses in the US, was chosen for the primary analysis based on the evidence available at the time from the pre-licensure RV1 and RV5 clinical trials. Intussusception was also assessed in 0–6 day and 0–29 day risk periods. Follow-up was censored permanently upon the earliest of: one year of age, health plan disenrollment, death, receipt of RV5 vaccination (RV1 and hIPV recipients only), receipt of RV1 vaccination (cIPV and hIPV recipients only), or last day of follow-up (30 November 2013 for RV1 and cIPV recipients and 31 March 2009 for hIPV recipients). Administration of Dose 2 of study vaccine within 60 days following administration of Dose 1 restarted follow-up for the subject for up to an additional 60 days.

### Sample size

As RV1 is administered according to a two-dose schedule, sample size was driven by age-month-specific incidence rates (IRs) of intussusception following Dose 1 and Dose 2 of RV1. It was estimated that a total of 55,700 RV1 recipients and up to 167,100 IPV recipients in each of the comparison groups were needed in order to have 80% power to detect a relative risk of 2.5 or higher during a 60-day risk period after both doses combined. This sample size estimate was based on a two-sided (alpha = 0.05) test (likelihood ratio test for two independent proportions), assuming the ratio of the cIPV recipients to RV1 recipients to be 3:1 and the cumulative incidence in the cIPV cohort during 60 days after the first 2 doses to be 13.2/100,000. Infants were identified from 2 claims databases (ORD and HIRD) in order to meet the sample size and study power requirements.

### Analysis

Infants were characterized by age, gender, US geographic region, and calendar quarter of cohort entry. The outcome analysis included medical record-confirmed events for intussusception, Kawasaki disease, and convulsion, all claims-identified events for acute LRTI, and claims and NDI-identified events for all-cause mortality. For the primary analysis, outcome IRs were calculated following each dose of study vaccine as the number of events divided by the sum of the person-time in that risk period. The exact confidence limit for the Poisson distribution was used to calculate 95% confidence intervals (CIs) for the IRs. Outcome IRs among RV1 recipients were compared to those among cIPV recipients and hIPV recipients with incidence rate ratios (IRRs) and corresponding 95% CIs obtained from Poisson regression models adjusted for age at specific vaccination, gender, dose-specific calendar quarter of vaccination, and database (ORD or HIRD). Study criterion for the absence of increased outcome risk was that the 95% CI of the IRR contained 1, or an upper limit below 2.5.

Several post-hoc analyses were also conducted to inform the interpretation of the planned analyses. A self-controlled case-series (SCCS) analysis of convulsion was conducted to further investigate the risk during the first 7 days following vaccination. This analysis compared within each group of recipients the IR of convulsion in the 0–7 day risk period following vaccination to the IR in the 15–30 day self-control period following vaccination. IRs and IRRs for acute LRTI were estimated in 0–6 and 0–29 day risk periods using the same methods as in the primary analysis. To further investigate these findings, a temporal cluster analysis of acute LRTI was conducted using SaTScan Software version 9.4.^22^ Clusters were explored among the RV1 and IPV recipients within 1 and 2-week time windows following each vaccine dose and the number of observed and expected observations inside the time windows were noted to calculate the likelihood of those observations. Under the null hypothesis, the observed events occur randomly following a uniform distribution according to a discrete Poisson model during the total observation period.^23^ The window with the maximum likelihood was the cluster least likely to be due to chance.

### Privacy and confidentiality

We obtained approval of the study protocol and a waiver of patient authorization from the New England Institutional Review Board and Privacy Board. All analyses were performed using appropriately de-identified data without access to personal identifying information.

## Results

A total of 57,931 RV1 recipients were accrued and matched to 173,384 cIPV recipients and 159,344 hIPV recipients (Table 2). Among all vaccine recipients, the mean age at cohort entry was 2 months, and 48% were female. The average length of follow-up among RV1, cIPV, and hIPV recipients was 98, 105, and 102 days, respectively, across both vaccine doses. Follow-up was censored permanently for 2.4% of RV1 recipients, 0.6% of cIPV recipients, and 2.3% of hIPV recipients due to receipt of a censoring vaccine, and for 13.1% of RV1 recipients, 13.8% of cIPV recipients, and 14.8% of hIPV recipients due to health plan disenrollment. Censoring due to reaching one year of age, the end of the study period, or the occurrence of claims-based death was rare among all vaccine recipients.

**Table 2.** Characteristics of RV1 and IPV recipients.

	RV1 N = 57,931	cIPV N = 173,384	hIPV N = 159,344
Infants with Dose 1 (n, %)^*^	57,931 (100.0)	173,324 (> 99.9)	159,344 (100.0)
Infants with Dose 2 (n, %)	42,097 (72.7)	149,944 (86.5)	129,741 (81.4)
Age at Cohort Entry (mean, SD) (Months)	2.0 (0.7)	2.0 (0.7)	2.0 (0.7)
Female (n, %)	27,929 (48.2)	83,528 (48.2)	77,128 (48.4)
Calendar Quarter of Cohort Entry (n, %)			
Q1	14,196 (24.5)	42,587 (24.6)	39,921 (25.1)
Q2	14,173 (24.5)	42,398 (24.5)	39,834 (25.0)
Q3	14,707 (25.4)	43,830 (25.3)	40,465 (25.4)
Q4	14,855 (25.6)	44,569 (25.7)	39,124 (24.6)
US Geographic Region (n, %)			
Northeast	8,461 (14.6)	18,093 (10.4)	22,171 (13.9)
South/Southeast	19,875 (34.3)	59,167 (34.1)	61,965 (38.9)
Midwest	17,101 (29.5)	51,046 (29.4)	41,114 (25.8)
West	12,494 (21.6)	45,078 (26.0)	34,094 (21.4)

Abbreviations: cIPV = concurrent IPV; hIPV = historical IPV; RV1 = human rotavirus vaccine; IPV = inactivated poliovirus vaccine; N = total number of infants; n, % = number / percentage of infants in a given category; Q = calendar quarter; SD = standard deviation.

* 60 concurrent IPV recipients entered the study on Dose 2 of vaccine.

Among RV1 and IPV recipients, 67 intussusception, 1,966 acute LRTI, 24 Kawasaki disease, and 593 convulsion events were identified in the claims data, and 69 mortality events were identified in the claims data or NDI. Medical records were obtained for 85.1%, 70.8%, and 76.9% of claims-identified intussusception, Kawasaki disease, and convulsion events, respectively (Table 3). Of the cases for which a medical record was obtained, 49.1% of intussusception cases, 58.9% of Kawasaki disease cases, and 45.2% of convulsion cases were confirmed.

**Table 3.** Summary of claims-identified and medical record-confirmed intussusception, kawasaki disease, and convulsion events among HRV and IPV recipients.

Outcome overall and by vaccine recipient	Claims-identified N	Medical record obtained^*^ n (%)	Medical record confirmed^†^ n (%)
Intussusception	67	57 (85.1)	28 (49.1)
RV1	7	7 (100.0)	4 (57.1)
cIPV	37	31 (83.8)	13 (41.9)
hIPV	23	19 (82.6)	11 (57.9)
Kawasaki disease	24	17 (70.8)	10 (58.9)
RV1	2	2 (100.0)	0 (0.0)
cIPV	14	10 (71.4)	5 (50.0)
hIPV	8	5 (62.5)	5 (100.0)
Convulsion	593	456 (76.9)	206 (45.2)
RV1	93	79 (84.9)	43 (54.4)
cIPV	254	204 (80.3)	85 (41.7)
hIPV	246	173 (70.3)	78 (45.1)

Abbreviations: cIPV = concurrent IPV; hIPV = historical IPV; RV1 = human rotavirus vaccine; IPV = inactivated poliovirus vaccine.

* Percentages are proportions based on the number of claims-identified events within each category.

† Percentages are proportions based on the number of medical records obtained within each category.

The IRRs during the 0–59 day risk period ranged from 0.36–2.25 for medical record-confirmed intussusception, 0.83–1.16 for acute LRTI, and 0.61 – 1.78 for all-cause mortality (Table 4). These IRRs were consistent with no increased outcome risk based on the corresponding 95% CIs. No medical record-confirmed intussusception events were identified among RV1 recipients in the 0–6 day risk period following any dose, and only one event was observed in the 0–29 day risk period following Dose 2. No medical record-confirmed Kawasaki disease events were identified among RV1 recipients. The IRRs of medical record-confirmed convulsion among RV1 recipients compared to cIPV and hIPV recipients were elevated following Dose 1 (2.07, 95% CI: 1.27 – 3.38 and 2.05, 95% CI: 1.24 – 3.38, respectively), but not following Dose 2 (1.35, 95% CI: 0.76 – 2.40 and 1.24, 95% CI: 0.69 – 2.21, respectively) (Table 4). In analyses of the individual databases, the IRR of medical record-confirmed convulsion following Dose 1 was elevated in the HIRD (RV1 vs. cIPV IRR: 2.58, 95% CI: 1.42 – 4.70; RV1 vs. hIPV IRR: 5.06, 95% CI: 2.36 – 10.81), but not in the ORD (RV1 vs. cIPV IRR: 1.32, 95% CI: 0.54 – 3.21); RV1 vs. hIPV IRR: 0.80, 95% CI: 0.35 – 1.83) (data not shown in a table).

**Table 4.** Incidence Rates (per 1,000 person-months) and Incidence Rate Ratios of Study Outcomes in the 0–59 Day Risk Period Following RV1 Vaccination Relative to IPV Vaccination.

	RV1			cIPV			hIPV			RV1 vs. cIPV	RV1 vs. hIPV
Dose	N	Person-Months	IR(95% CI)	N	Person-Months	IR(95% CI)	N	Person-Months	IR (95% CI)	IRR^*^(95% CI)	IRR^*^(95% CI)
Medical Record-Confirmed Intussusception											
Any	4	187,108	0.021	13	599,928	0.022	11	533,379	0.021	0.97	1.04
			(0.006–0.055)			(0.012 –0.037)				(0.010 –0.037)	(0.31 –2.96)	(0.33 –3.29)
1	3	107,284	0.028	4	321,070	0.012	5	291,405	0.017	2.25	1.65	
			(0.006–0.082)			(0.003 –0.032)				(0.006 –0.040)	(0.50 –10.05)	(0.39 –6.92)
2	1	79,824	0.013	9	278,858	0.032	6	241,973	0.025	0.36	0.48	
			(0.000–0.070)			(0.015 –0.061)				(0.009–0.054)	(0.05 –2.85)	(0.06 –4.00)
Hospitalization due to Acute LRTI												
Any	284	187,108	1.518	792	599,928	1.320	890	533,379	1.669	1.13	0.87	
			(1.346 –1.705)			(1.230 –1.415)				(1.561 –1.782)	(0.99 –1.30)	(0.76 –1.00)
1	182	107,284	1.696	491	321,070	1.529	585	291,405	2.008	1.11	0.83	
			(1.459 –1.962)			(1.397 –1.671)				(1.848 –2.177)	(0.94 –1.32)	(0.70 –0.98)
2	102	79,824	1.278	301	278,858	1.079	305	241,973	1.260	1.16	0.96	
			(1.042 –1.551)			(0.961 –1.208)				(1.123 –1.410)	(0.93 –1.46)	(0.77 –1.21)
Medical Record-Confirmed Convulsion												
Any	43	187,108	0.230	85	599,928	0.142	78	533,379	0.146	1.75	1.62	
			(0.166 –0.310)			(0.113 –0.175)				(0.116 –0.183)	(1.21 –2.54)	(1.12 –2.36)
1	27	107,284	0.252	39	321,070	0.121	36	291,405	0.124	2.07	2.05	
			(0.166 –0.366)			(0.086 –0.166)				(0.087 –0.171)	(1.27 –3.38)	(1.24 –3.38)
2	16	79,824	0.200	46	278,858	0.165	42	241,973	0.174	1.35	1.24	
			(0.115 –0.326)			(0.121 –0.220)				(0.125 –0.235)	(0.76 –2.40)	(0.69 –2.21)
All-Cause Mortality												
Any	9	187,104	0.048	27	599,921	0.045	33	533,366	0.062	1.05	0.75	
			(0.022 –0.091)			(0.030 –0.065)				(0.043 –0.087)	(0.50 –2.24)	(0.36 –1.57)
1	5	107,281	0.047	19	321,066	0.059	22	291,397	0.075	0.79	0.61	
			(0.015 –0.109)			(0.036 –0.092)				(0.045 –0.114)	(0.29 –2.11)	(0.23 –1.61)
2	4	79,823	0.050	8	278,855	0.029	11	241,969	0.045	1.78	1.06	
			(0.014 –0.128)			(0.012 –0.057)				(0.023 –0.081)	(0.53 –5.92)	(0.34 –3.33)

Abbreviations: RV1 =human rotavirus vaccine; IPV = inactivated poliovirus vaccine; cIPV = concurrent IPV; hIPV = historical IPV; N = number of cases; IR = incidence rate; CI = confidence interval; IRR = incidence rate ratio; LRTI = lower respiratory tract infection.

* IRRs were adjusted for age at specific vaccination, gender, dose-specific calendar quarter of vaccination, and database (ORD or HIRD).

In the post-hoc SCCS analysis, the IRR of medical record-confirmed convulsion among RV1 recipients in the 0–7 day risk period versus the 15–30 day self-control period was 2.40 (95% CI: 0.73 – 7.86) following Dose 1 (Supplemental Table 1). In the post-hoc analysis of acute LRTI, the IRR among RV1 recipients compared to cIPV recipients in the 0–6 day risk period was slightly elevated following Dose 1 (1.66, 95% CI: 1.03–2.68) but not following Dose 2 (1.01, 95% CI: 0.41–2.49) (Supplemental Table 2). No significant temporal clusters of acute LRTI cases were observed among RV1 recipients (Supplemental Table 3).

## Discussion

This post-licensure observational study evaluated the risk of 5 safety outcomes among 57,931 commercially-insured infants receiving RV1 during the first 5 years following post-marketing approval in the US. To our knowledge, this study is the first to assess the risk of intussusception and other safety outcomes over a 60-day risk period following each dose of RV1 administration.

No evidence of an increased risk of medical record-confirmed intussusception was observed during the 0–59 day risk period following vaccination. Later post-licensure studies of RV1 have reported an increased risk of intussusception following rotavirus vaccination.^24-27^ The most recent publications have reported an increased risk of intussusception in the first 7 days following rotavirus vaccination, especially after the first dose.^28-30^ At the time this study was designed, an increased risk for intussusception following RV1 or RV5 in the first 7 days following vaccination was not evident based on pre-licensure clinical trials of RV1 or RV5 or in early post-marketing studies of RV5. Additionally, the detection of an increased risk during the first 7 days post-vaccination would have required a large sample size that would not have been accrued during the early post-approval years as a sufficient number of RV1 doses was not administered. However, the intussusception findings from the 0–59 day risk window among this study population of infants with commercial health plan insurance informs the safety profile of RV1 in a population which would not be represented in the other publications, which used data from active surveillance systems. We observed no confirmed cases of intussusception in the 0–6 day risk period following RV1 administration, which is consistent with the expected number of cases (<0.5) given the small number of RV1 doses administered during the study period. This study was not powered to detect an increased risk of intussusception in the first week following RV1 administration, and our findings in the 0–6 day risk period therefore do not confirm or refute those of the previously published studies. A study powered to detect intussusception risk within the first week following RV1 administration within a population of commercially-insured infants would provide additional information.

No evidence of an increased risk of acute LRTI was observed in the 0–59 day risk period following RV1 administration. A slight increased risk of acute LRTI was observed among RV1 recipients relative to cIPV recipients in the 0–6 day risk period following Dose 1, but no significant temporal clusters among RV1 recipients were detected. To date, no published studies have identified an increased risk of acute LRTI following RV1 administration.

The occurrence of Kawasaki disease in this study population was rare, with no medical record-confirmed events observed among RV1 recipients. This finding is consistent with the findings of a safety study of RV5 among commercially-insured infants.^19^ To date, no published studies have identified an increased risk of Kawasaki disease following RV1 administration.

A 2-fold increased risk of medical record-confirmed convulsion was observed following Dose 1 of RV1, but was not observed following Dose 2. In the post-hoc SCCS analysis, which was conducted to further investigate the timing of the convulsion events within the 0–59 day risk period, the rate of convulsion was approximately 2 times higher in the 0–7 day risk period following RV1 Dose 1 compared to the 15–30 day self-control period. The estimate in the SCCS analysis was less precise due to the small number of cases available and should be interpreted cautiously. Several factors should be considered when interpreting the convulsion findings. The increased risk of medical record-confirmed convulsion among RV1 recipients following Dose 1 in comparison to the IPV recipients was observed in only one of the data sources. Also, the IR of convulsion among RV1 recipients following Dose 1 was low (0.25 per 1,000 person-months) and similar to the IR following Dose 2 (0.20 per 1,000 person-months). Recent studies report that children receiving a full course of rotavirus vaccination (3 doses of RV5 or 2 doses of RV1) have a significantly lower risk of seizures requiring emergency care attendance or hospitalization compared to children not receiving rotavirus vaccination.^31-33^ The likely mechanism of this protective effect is the direct role of vaccination in the prevention of rotavirus illness and the associated gastrointestinal and central nervous system complications.^34-37^ Although an increased risk of convulsion has been demonstrated with concomitant administration of some vaccines,^38^ this is unlikely to be an explanation for our findings as we do not expect rates of concomitant vaccination to be different between the RV1 and IPV recipients. To date, no other published studies have identified an increased risk of convulsion following RV1 administration. The findings from the current study are not sufficient to demonstrate a causal relationship between the incidence of convulsion and RV1 administration.

No evidence of an increased risk of all-cause mortality was observed in the 0–59 day risk period following RV1 administration. A 1.78-fold increased risk of mortality compared to cIPV recipients was observed following Dose 2. The wide 95% CI (0.53 – 5.92) surrounding this estimate is indicative of the small number of events. The risk of mortality following Dose 1, on the other hand, was lower than 1 (IRR: 0.79, 95% CI: 0.29 – 2.11). Additionally, an elevated risk of mortality was not observed among RV1 recipients compared to hIPV recipients following any vaccine dose. These findings indicate that elevated risk observed in RV1 recipients compared to cIPV recipients following Dose 2 was consistent with chance. Our all-cause mortality findings are consistent with those reported in a safety study of RV5 among commercially-insured infants.^19^ To date, no published studies have identified an increased risk of all-cause mortality following RV1 administration.

The findings of this study should be interpreted within the context of several study design considerations. The small number of RV1 recipients, combined with the rarity of intussusception, Kawasaki disease, and mortality cases in the overall study population led to limited power for the analyses of these 3 outcomes. Several factors contributed to the small number of RV1 recipients. RV1 was licensed in the US in 2008, 2 years after RV5, so the level of RV1 uptake during our study period had not reached the level of RV5 uptake. RV1 uptake was also affected in 2010 by the temporary suspension of its use following discovery of contamination with porcine circovirus fragments. A similar contamination of RV5 was subsequently announced, but the use of RV5 continued. A study which assessed the effect of this suspension on the utilization of RV1 and RV5 observed that RV1 use decreased and switching to RV5 increased.^39^ RV1, RV5, and IPV vaccinations were identified on the basis of CPT codes recorded on health insurance claims submitted by providers, which were assumed to reflect the vaccine product used. In a validation sub-study using these data that compared rotavirus vaccine codes against medical records, the RV1 and RV5 codes were accurate 89% and 87% of the time, so that misclassification of rotavirus vaccine product was unlikely to have an impact on our findings.^40^

Findings from the secondary 0–7 and 0–29 day risk period analyses, particularly for intussusception, should be interpreted with caution due to the small sample available for this analysis. RV1, cIPV, and hIPV recipients had three different vaccine censoring criteria that avoided undesirable conflation of exposure but introduced the potential for selection bias in scenarios where the rate of the outcome varies over the risk periods. IRs of acute LRTI were therefore calculated without the vaccine censoring criteria applied in a sensitivity analysis, leading to the gain of a small number of acute LRTI cases in the additional person-time. This gave rise to IRs that were virtually identical to those with the vaccine censoring criteria applied. Additionally, as 88% of cIPV recipients received RV5, the IR of intussusception among RV1 recipients was compared to the IR among cIPV recipients who also received RV5. The findings were similar to the primary analysis, with IRRs in the 0–59 day risk period of 1.00 (95% CI: 0.32 – 3.15) following any vaccine dose, 2.00 (95% CI: 0.45 – 8.93) following Dose 1, and 0.42 (95% CI: 0.05 – 3.41) following Dose 2 (data not shown in a table).

Our medical record review process for intussusception, Kawasaki disease, and convulsion ensured that the events included in the analysis met a clinical definition and were not merely claims bearing the diagnosis, as might happen with a service performed to rule out the condition. However, 23% of the medical records requested could not be obtained due to provider refusal or non-response. These unattained records may have included some true events that were not retained in the analyses, which may have biased our effect estimates. We conducted a sensitivity analysis where these non-obtained medical records, in addition to the medical record-confirmed cases, were included in the IR and IRR estimates for intussusception, Kawasaki disease, and convulsion. The estimates in the sensitivity analysis were similar to the estimates including only the medical record-confirmed cases (data not shown). Acute LRTI and all-cause mortality were not confirmed through medical record review, as it was possible to operationalize these outcome definitions strictly on the basis of claims for medical services and also from the NDI for mortality. The performance of the outcome definition for acute LRTI was evaluated in the ORD for a random sample of patients by manual review by a clinician of claims for all medical services occurring within the hospitalization that gave rise to the acute LRTI event. This review indicated that the claims definition for acute LRTI accurately identified outcomes.

While claims data are valuable for the examination of health care outcomes, treatment patterns, health care resource utilization, and costs, all claims databases have certain inherent limitations, because the claims are collected for the purpose of payment and not research. Presence of a diagnosis code on a medical claim does not always indicate the presence of a disease, as the diagnosis code may be included as rule-out criteria rather than actual disease. The infants in this study population had health insurance coverage through a parent or guardian who is employed, and are likely to receive their vaccinations through a provider participating in the health insurance plan, rather than through clinics which provide free or reduced-cost vaccinations. The findings of this study may therefore not be generalizable to infants in the US who do not have health insurance coverage and therefore have a different pattern of medical care.

This study has several strengths. The analysis was conducted on a study population identified from 2 large health insurance databases, making for a well-powered analysis for the outcomes of acute LRTI and convulsion. Additionally, 2 comparator groups (concurrent and historical IPV recipients) were used to allow for control of various factors that could not be achieved by the use of a single comparison group. Although the majority of the cIPV recipients received RV5 due to routine vaccination recommendations, this cohort provided the ability to control for possible changes in diagnosis patterns over time. The historical IPV cohort provided a cohort of IPV-exposed infants that was not exposed to any rotavirus vaccine due to the unavailability of rotavirus vaccines during that time frame. The comparator cohorts were similar to each other with respect to their age and sex distributions. The results from both comparison groups should be interpreted within the context of these factors.

In conclusion, this large observational cohort study supports the favorable safety profile of RV1 vaccination. The increased convulsion risk following RV1 Dose 1 is inconclusive due to the heterogeneity of the results between the 2 claims databases. However, continued monitoring for safety signals through routine surveillance is needed to ensure vaccine safety.

## Trade/service marks

Rotarix is a trademark of the GSK Group of companies. RotaTeq is a trademark of Merck & Co., Inc. Rotashield is a trademark of Wyeth Laboratories. SaTScan is a trademark of Martin Kulldorff. The SaTScan software was developed under the joint auspices of (i) Martin Kulldorff, (ii) the National Cancer Institute, and (iii) Farzad Mostashari of the New York City Department of Health and Mental Hygiene. The HealthCore Integrated Research Database (HIRD) is a service mark of HealthCore, Inc.

## Supplementary Material

KHVI_A_1450123_Supplemental.docx
