# Supplementary material for: Safety study of live, oral human rotavirus vaccine: A cohort study in United States health insurance plans
Source: Hum Vaccin Immunother. 2018 Apr 13;14(7):1782–90. doi: 10.1080/21645515.2018.1450123 (PMC6067866; doi:10.1080/21645515.2018.1450123)
Supplement: KHVI_A_1450123_Supplemental.docx [file khvi-14-07-1450123-s001.docx]

Supplemental Table 1. Self-Controlled Case Series Analysis for Convulsion following vaccination with RV1 or IPV

| Cohort | Vaccine Dose | Period^*^ | N | Person-Months | IRR | 95% CI | P-Value |
| --- | --- | --- | --- | --- | --- | --- | --- |
| RV1 | Any | Risk | 8 | 3.7 | 2.67 | 0.93 – 7.69 | 0.069 |
|  |  | Control | 6 | 7.4 | ─ | ─ |  |
|  | 1 | Risk | 6 | 2.9 | 2.40 | 0.73 – 7.86 | 0.148 |
|  |  | Control | 5 | 5.8 | ─ | ─ |  |
|  | 2 | Risk | 2 | 0.8 | 4.00 | 0.36 – 44.12 | 0.258 |
|  |  | Control | 1 | 1.6 | ─ | ─ |  |
| cIPV | Any | Risk | 14 | 7.9 | 1.74 | 0.85 – 3.56 | 0.131 |
|  |  | Control | 16 | 15.7 | ─ | ─ |  |
|  | 1 | Risk | 8 | 3.4 | 3.15 | 1.03 – 9.64 | 0.044 |
|  |  | Control | 5 | 6.7 | ─ | ─ |  |
|  | 2 | Risk | 6 | 4.5 | 1.09 | 0.40 – 2.95 | 0.864 |
|  |  | Control | 11 | 8.9 | ─ | ─ |  |
| hIPV | Any | Risk | 10 | 7.6 | 1.05 | 0.49 – 2.26 | 0.896 |
|  |  | Control | 19 | 15.2 | ─ | ─ |  |
|  | 1 | Risk | 5 | 4.7 | 0.77 | 0.27 – 2.16 | 0.618 |
|  |  | Control | 13 | 9.5 | ─ | ─ |  |
|  | 2 | Risk | 5 | 2.9 | 1.67 | 0.51 – 5.46 | 0.399 |
|  |  | Control | 6 | 5.8 | ─ | ─ |  |

Abbreviations: CI = confidence interval; cIPV = concurrent IPV cohort; hIPV = historical IPV cohort; RV1 = Human Rotavirus Vaccine cohort; IPV = Inactivated Poliovirus Vaccine; IRR = incidence rate ratio; N = number of cases

^*^Risk period: 0-7 days following vaccination; Control period = 15-30 days following vaccination

| Supplemental Table 2. Incidence Rates (per 1,000 person-months) and Incidence Rate Ratios of Hospitalization due to Acute LRTI in the 0-6 and 0-29 Day Risk Periods Following RV1 Vaccination Relative to IPV Vaccination | | | | | | | | | | | |
| --- | --- | --- | --- | --- | --- | --- | --- | --- | --- | --- | --- |
|  | RV1 | | | cIPV | | | hIPV | | | RV1 vs.  cIPV | RV1 vs.  hIPV |
| Dose | N | Person-Months | IR  (95% CI) | N | Person-Months | IR  (95% CI) | N | Person-Months | IR  (95% CI) | IRR^*^  (95% CI) | IRR^*^  (95% CI) |
| 0-6 Day Risk Period | | | | | | | | | | | |
| Any | 32 | 22,911 | 1.397  (0.955 – 1.972) | 69 | 74,034 | 0.932  (0.725 – 1.180) | 93 | 66,146 | 1.406  (1.135 – 1.722) | 1.46  (0.96 – 2.22) | 0.96  (0.64 – 1.44) |
| 1 | 26 | 13,268 | 1.960  (1.280 – 2.871) | 47 | 39,692 | 1.184  (0.870 – 1.575) | 70 | 36,447 | 1.921  (1.497 – 2.427) | 1.66  (1.03 – 2.68) | 1.00  (0.64 – 1.57) |
| 2 | 6 | 9,643 | 0.622  (0.228 – 1.354) | 22 | 34,342 | 0.641  (0.401 – 0.970) | 23 | 29,699 | 0.774  (0.491 – 1.162) | 1.01  (0.41 – 2.49) | 0.84  (0.34 – 2.08) |
| 0-29 Day Risk Period | | | | | | | | | | | |
| Any | 154 | 96,679 | 1.593  (1.351 – 1.865) | 432 | 312,465 | 1.383  (1.255 – 1.519) | 497 | 277,976 | 1.788  (1.634 – 1.952) | 1.15  (0.96 – 1.38) | 0.85  (0.71 – 1.02) |
| 1 | 104 | 55,964 | 1.858  (1.518 – 2.252) | 255 | 167,473 | 1.523  (1.341 – 1.721) | 331 | 152,843 | 2.166  (1.939 – 2.412) | 1.22  (0.97 – 1.54) | 0.84  (0.68 – 1.05) |
| 2 | 50 | 40,716 | 1.228  (0.911 – 1.619) | 177 | 144,992 | 1.221  (1.048 – 1.414) | 166 | 125,134 | 1.327  (1.132 – 1.544) | 1.00  (0.73 – 1.38) | 0.89  (0.65 – 1.22) |
| Abbreviations: CI = confidence interval; cIPV = concurrent IPV; hIPV = historical IPV; RV1 = human rotavirus vaccine; IPV = inactivated poliovirus vaccine; IR = incidence rate; IRR = incidence rate ratio; LRTI = lower respiratory tract infection; N = number of cases  ^*^IRRs were adjusted for age at specific vaccination, gender, dose-specific calendar quarter of vaccination, and database (ORD or HIRD). | | | | | | | | | | | |

Supplemental Table 3. Temporal Cluster Analysis^*^ of Acute LRTI Hospitalization in 0-59 Days Following Vaccination

with RV1 or IPV

|  | Temporal Window | Vaccine Dose | Time Frame of Most Likely Cluster^†^ | O/E Cases | O/E Ratio | RR | P-value |
| --- | --- | --- | --- | --- | --- | --- | --- |
| RV1 | 1 Week | Any | 12 to 14 | 23/14.20 | 1.62 | 1.67 | 0.810 |
|  |  | Dose 1 | 3 to 8 | 29/18.20 | 1.59 | 1.71 | 0.520 |
|  |  | Dose 2 | 38 to 42 | 15/8.50 | 1.76 | 1.90 | 0.760 |
|  | 2 Weeks | Any | 3 to 14 | 75/56.80 | 1.32 | 1.44 | 0.495 |
|  |  | Dose 1 | 3 to 14 | 51/36.40 | 1.40 | 1.56 | 0.481 |
|  |  | Dose 2 | 38 to 42 | 15/8.50 | 1.76 | 1.90 | 0.760 |
| cIPV | 1 Week | Any | 9 to 14 | 110/79.20 | 1.39 | 1.45 | 0.041 |
|  |  | Dose 1 | 33 to 34 | 29/16.37 | 1.77 | 1.82 | 0.248 |
|  |  | Dose 2 | 18 to 22 | 43/25.08 | 1.71 | 1.83 | 0.043 |
|  | 2 Weeks | Any | 9 to 22 | 238/184.80 | 1.29 | 1.41 | 0.003 |
|  |  | Dose 1 | 33 to 34 | 29/16.37 | 1.77 | 1.82 | 0.301 |
|  |  | Dose 2 | 9 to 22 | 104/70.23 | 1.48 | 1.73 | 0.002 |
| hIPV | 1 Week | Any | 4 to 10 | 148/103.83 | 1.43 | 1.51 | 0.002 |
|  |  | Dose 1 | 4 to 10 | 104/68.25 | 1.52 | 1.64 | 0.002 |
|  |  | Dose 2 | 8 to 13 | 47/30.50 | 1.54 | 1.64 | 0.206 |
|  | 2 Weeks | Any | 4 to 13 | 209/148.33 | 1.41 | 1.53 | 0.001 |
|  |  | Dose 1 | 4 to 14 | 156/107.25 | 1.45 | 1.62 | 0.001 |
|  |  | Dose 2 | 8 to 18 | 77/55.92 | 1.38 | 1.50 | 0.187 |

Abbreviations: cIPV = concurrent IPV; E = expected; hIPV = historical IPV; RV1 = human rotavirus vaccine; IPV = inactivated poliovirus vaccine; LRTI = lower respiratory tract infection; O = observed; RR = relative risk

^*^The temporal cluster analysis was conducted using SaTScan Software version 9.4.

^†^Days following vaccine dose
